# Protective effect of Qingluotongbi formula against *Tripterygium wilfordii* induced liver injury in mice by improving fatty acid β-oxidation and mitochondrial biosynthesis

**DOI:** 10.1080/13880209.2022.2157842

**Published:** 2022-12-21

**Authors:** Jie Zhou, Ming Li, Zhichao Yu, Changqing Li, Lingling Zhou, Xueping Zhou

**Affiliations:** a The First Clinical Medical College, Nanjing University of Chinese Medicine, Nanjing, China; b The First Affiliated Hospital of Anhui University of Chinese Medicine, Hefei, China; c Jiangsu Provincial Key Laboratory of Pharmacology and Safety Evaluation of Material Medical, School of Pharmacy, Nanjing University of Chinese Medicine, Nanjing, China

**Keywords:** traditional Chinese medicine, hepatotoxicity, compound compatibility

## Abstract

**Context:**

Qingluotongbi formula (QLT) is a Chinese medicine compound consisting of *Tripterygium wilfordii* Hook. f. (Celastraceae, TW), *Panax notoginseng* (Burkill) F.H.Chen (Araliaceae, PN), *Rehmannia glutinosa* (Gaertn.) DC. (Orobanchaceae, RG), *Sinomenium acutum* (Thunb.) Rehder & E.H. Wilson (Menispermaceae, SA), and *Bombyx mori* L. (Bombycidae, BM).

**Objective:**

This study investigated the protective effect and possible mechanism of QLT against TW-induced liver injury in mice.

**Materials and methods:**

To establish the model of TW-induced liver injury in mice, C57BL/6J mice were randomly divided into 4 groups: control group, low-dose TW group, middle-dose TW group, and high-dose TW group. To observe the effects of QLT and its individual ingredients against TW-induced liver injury, C57BL/6J mice were randomly divided into 7 groups: control group, TW group, QLT group, PN group, RG group, SA group, BM group.

After administration for 7 days, C57BL/6J mice were tested for biochemical indicators and liver pathological changes. Then, we evaluated the mitochondrial function and analysed the gene and protein expression related to the peroxisome proliferator-activated receptor alpha (PPARα)/peroxisome proliferator-activated receptor gamma coactivator-1 alpha (PGC-1α) pathway by quantitative real-time PCR (qRT-PCR) and Western blotting.

**Results:**

Compared with the control group (0.30 ± 0.35), TW significantly increased mice liver histological score (L, 0.95 ± 1.14; M, 1.25 ± 1.16; H, 4.00 ± 1.13). QLT and its ingredients significantly improved the pathology scores (CON, 0.63 ± 0.74; TW, 4.19 ± 1.53; QLT, 1.56 ± 0.62; PN, 1.94 ± 0.68; RG, 2.75 ± 1.39; SA, 4.13 ± 0.99; BM, 4.13 ± 0.99). Western blot and qRT-PCR analysis revealed that QLT and its ingredients reversed TW-induced suppression of PPARα/PGC1-α pathway.

Discussion and conclusions: These findings provide valuable information for compound compatibility studies and TW clinical applications.

## Introduction

Current pharmacological studies have shown that *Tripterygium wilfordii* Hook. f. (Celastraceae) (TW), a widely used traditional Chinese medicine in autoimmune and inflammatory diseases, possesses prominent anti-inflammatory and immunosuppressive effect (Zhang et al. 2021). However, some side effects, especially hepatotoxicity, limit its further development in the clinic. The molecular mechanisms of TW hepatotoxicity have not been fully defined.

It is an urgent unmet need to elucidate TW hepatotoxicity mechanisms and to find effective methods for mitigating toxicity. Compound compatibility is the most practical and effective means in clinical application and can reduce the toxicity of TW (Cao et al. 2015; Song et al. 2020). Qingluotongbi formula (QLT) consists of TW and other four ingredients: *Panax notoginseng* (Burkill) F.H.Chen (Araliaceae, PN), *Rehmannia glutinosa* (Gaertn.) DC. (Orobanchaceae, RG), *Sinomenium acutum* (Thunb.) Rehder & E.H. Wilson (Menispermaceae, SA), and *Bombyx mori* L. (Bombycidae, BM). QLT originates from the great TCM master Prof. Zhou Zhongying in clinical practice, and no obvious adverse reactions were observed during the treatment (Zhou et al. 2004). Based on this, Prof. Zhou Xueping proposed the ‘Heterogeneous Restriction’ (Yilei Xiangzhi) theory and conducted research on TW compatibility for detoxification. We found that QLT can lessen TW hepatotoxicity and when all five ingredients were used together, the best improvements were seen. Therefore, we speculated that the combination of various ingredients in the formula had a synergistic effect, thus enhancing the detoxification effect.

The liver is the central metabolic organ of lipid metabolism. Abnormal lipid metabolism is a general feature of TW-induced liver injury, manifesting as increased free and esterified fatty acids levels (Liu et al. 2021). Fatty acids are oxidized mainly within mitochondria to provide energy for cellular function. Numerous dysfunctions can be caused by impaired fatty acid oxidation, such as abnormal energy metabolism and hepatocyte vulnerability to oxidative stress (Liu et al. 2015). Our previous findings suggest that TW-indued liver injury mechanisms involve lipid anomalies (Xie et al. 2016). QLT and its ingredients can improve mitochondrial function (Zhou et al. 2018), reduce oxidative stress (Feng et al. 2019), restore lipid homeostasis (Yu et al. 2022), and thus mitigate TW hepatotoxicity.

Transcriptional regulation is an important way to regulate metabolic processes (Desvergne et al. 2006). Peroxisome proliferator-activated receptor alpha (PPARα), a member of nuclear receptor superfamily, is the master regulator of energy metabolism and lipid catabolism in the liver (Kersten and Stienstra 2017). Previous studies showed that the suppression of PPARα is linked to the hepatotoxic mechanisms of *Tripterygium* glycosides tablets and *Tripterygium wilfordii* tablets, two preparations of TW (Dai et al. 2022). PPARα activation can alleviate triptolide-induced liver injury in mice (Hu et al. 2019). The transcriptional coactivator peroxisome proliferator-activated receptor gamma coactivator-1 alpha (PGC-1α) controls multiple hepatic metabolic pathways (Duan et al. 2022), and acts as a coactivator of PPARα to coordinately regulate the transcription of genes related to fatty acid oxidation and mitochondrial biogenesis (Vega et al. 2000). PPARα/PGC-1α pathway plays a central role in the regulation of cellular metabolism (Cheng et al. 2018). The activation of PPARα/PGC-1α can improve mitochondrial function (Kelly and Scarpulla 2004) and reduce lipotoxicity by enhancing lipid metabolism (Kim et al. 2022). We therefore speculate that the hepatic protective effect of QLT is probably related to the PPARα/PGC-1α pathway.

We previously discovered that QLT can reduce TW-induced liver damage and lipid metabolic disorders, but the specific mechanisms have not yet been demonstrated. In this study, we examined whether QLT could exert its protective effect by improving mitochondrial function and enhancing fatty acid oxidation through the PPARα/PGC-1α pathway.

## Materials and methods

### Preparation of the aqueous extracts

TW was purchased from Jiangsu General Pharmaceutical Co., Ltd. (batch number: 201403, Taizhou, China); PN, RG, SA and BM were purchased from Bozhou Medicine Company (batch number: 131202, Anhui, China), and all the ingredients were identified by the School of Pharmacy, Nanjing University of Chinese Medicine (Nanjing, China).

**Figure 1.**
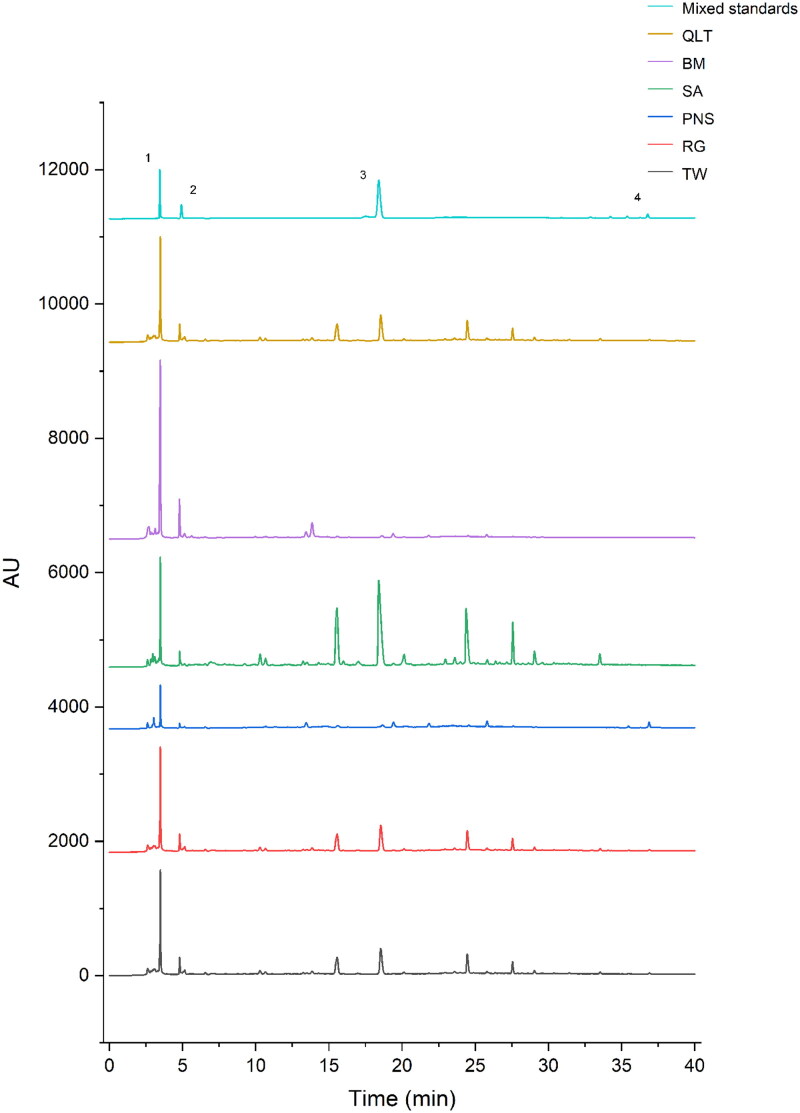
Fingerprint analysis by high-performance liquid chromatography (HPLC). (1) Triptolide; (2) Catalpol; (3) Sinomenine; (4) Notoginseng total saponins.

To prepare for TW group (TW), QLT group (TW + PN + RG + SA + BM), PN group (TW + PN), SA group (TW + SA), RG group (TW + RG), and BM group (TW + BM), all crude drugs were extracted twice with boiling water for 1.5 h and 1 h each time (1:11, w/v, and then 1:7, w/v), and filtered twice through two layers of gauze. We used high performance liquid chromatography to analyse the main active ingredients of Qingluotongbi recipe (Figure 1).

### Animals and treatment

Female C57BL/6J mice (6–8 weeks old, 18–22 g) were obtained from Qinglongshan animal breeding farm (Nanjing, China) (animal license number: SYXK (Su): 2017-0001). The animal study protocols were approved by the Ethics Review Committee for Animal Experimentation of Nanjing University of Chinese Medicine (animal ethics number: 201906A041).

To establish the model of TW-induced liver injury in mice, 40 C57BL/6J mice were randomly divided into 4 groups with 10 mice per group: blank control group (normal saline), low-dose TW group (75 g/70 kg/d), middle-dose TW group (150 g/70 kg/d), and high-dose TW group (300 g/70 kg/d).

To observe the effects of QLT and its individual ingredients against TW-induced liver injury, 56 C57BL/6J mice were randomly divided into 7 groups with 8 mice per group: blank control group (normal saline), TW group (300 g/70 kg/d), QLT group (343 g/70 kg/d), PN group (303 g/70 kg/d), RG group (315 g/70 kg/d), SA group (315 g/70 kg/d), BM group (310 g/70 kg/d).

Each group received consecutive intragastric administration for 7 days and was monitored daily for body weight and general condition. Mice were sacrificed 12 h after the last treatment, and liver and serum samples were collected for subsequent analysis.

### Biochemical assays

Blood was taken from mice by retro-orbital bleeding, and after standing at room temperature for 1 h, centrifuged at 4 °C, 3000 rpm for 5 min to collect the upper serum. Serum ALT, AST and LDH levels were measured using detection kits (Jiancheng, Nanjing, China) according to the manufacturer’s instructions.

Mouse livers were homogenized by a glass homogenizer and protein concentrations were determined using the BCA protein assay kit (Beyotime, Shanghai, China). MDA and NEFA in liver homogenate were detected according to the MDA detection kit (Beyotime, Shanghai, China) and free fatty acid detection kit (Jiancheng, Nanjing, China).

### Hematoxylin-eosin staining

Freshly collected livers were fixed in 4% paraformaldehyde, paraffin embedded, and sectioned, then stained with hematoxylin-eosin (H&E), and images were collected and scored under light microscopy (Leica DM5000B, Leica camera, Wetzler, Germany) (Brunt 2000; Kleiner et al. 2005).

### Oil Red O staining

The liver sections were fixed with 4% paraformaldehyde, washed with 60% isopropyl alcohol for 2 min, stained with Oil Red O (ORO) working solution for 10 min, immersed in 60% isopropyl alcohol again for colour separation, counterstained with hematoxylin after rinsed in distilled water, and the images were collected under light microscopy (Leica DM5000B, Leica camera, Wetzler, Germany).

### Transmission electron microscope observation

Fresh liver sections were fixed in 3% buffered glutaraldehyde, and then observed with transmission electron microscope (TEM) (H7700, Hitachi, Tokyo, Japan). Semi-quantitative analysis of images was performed with reference to the Flameng’s score (Flameng et al. 1980).

### Isolation of mitochondria

Mitochondria were isolated from mouse livers following the instructions of the mitochondrial isolation kit (Beyotime, Shanghai, China). Briefly, the homogenate was centrifuged at 1000 *g* for 5 min at 4 °C and the supernatant was centrifuged at 12,000 *g* for 10 min at 4 °C to isolate mitochondria. Then mitochondria were resuspended with wash buffer, centrifuged at 12,000 *g* for 10 min at 4 °C for high-purity mitochondria. Mitochondrial protein concentration was determined by BCA detection kit (Beyotime, Shanghai, China).

### Oxygen consumption rate measurement

The Clark-type oxygen electrode method was used to detect mitochondrial oxygen consumption. In brief, 100 μL of freshly prepared liver mitochondria were mixed with the reaction medium preheated at 25 °C, and 20 μL of 0.5 M disodium succinate was added to determine the ADP-free oxygen consumption rate (OCR) (ST4). Then 20 μL of 100 mM ADP was added to determine the OCR again (ST3). The respiratory control rate (RCR) was the rate of ST3 to ST4. The P/O ratio (P/O) was the ratio of the added amount of ADP to the ST3 oxygen consumption.

### Mitochondrial DNA quantitation

Mouse liver tissue DNA was extracted using the DNA extraction kit (Tiangen, Beijing, China), and the relative level of mitochondrial DNA (mtDNA) copy number was determined by quantitative real-time PCR (qRT-PCR) with the following primers: Rps18 Forward: 5′-AGGATGTGAAGGATGGGAAG-3′, Reverse: 5′-TTCTTCAGCCTCTCCAGGTC-3′; Cox2 Forward: 5′-TCATAATTGCCCTCCCCTCTC-3′, Reverse: 5′-ACTTCTAGCAGTCGTAGTTCACCAG-3′. Data are shown as fold change, defined by 2^‐ΔΔCT^.

### Quantitative real-time PCR

Total RNA was extracted with Trizol (Ambion, Austin, TX, USA), reverse transcribed to cDNA and qRT-PCR was performed according to the manufacturer’s instructions (Applied Biological Materials, Richmond, BC, Canada). The results were normalized to GAPDH expression. Data are shown as fold change, defined by 2^‐ΔΔCT^. The primers are described in Table 1.

**Table 1.** Primer sequence list.

Gene	Forward primer	Reverse primer
*Gapdh*	AGGTCGGTGTGAACGGATTTG	TGTAGACCATGTAGTTGAGGTCA
*Pparα*	GAGCTGCAAGATTCAGAAGAAG	GAATCTTTCAGGTCGTGTTCAC
*Cpt1α*	CTACATCACCCCAACCCATATT	GATCCCAGAAGACGAATAGGTT
*Ppargc1α*	GGATATACTTTACGCAGGTCGA	CGTCTGAGTTGGTATCTAGGTC
*Tfam*	GTGAGCAAGTATAAAGAGCAGC	CTGAACGAGGTCTTTTTGGTTT
*Nrf1*	GTTGCCCAAGTGAATTACTCTG	TCGTCTGGATGGTCATTTCAC
*Atp5f1α*	CTGCCTTACCAGTCATTGAAAC	TCCGTCAGTGATGGAAATAACA
*Cyt-c*	AAATCTCCACGGTCTGTTCG	TGCCCTTTCTCCCTTCTTC

### Western blotting

Total protein was extracted and quantified from liver tissue homogenates for western blotting. Primary antibodies included PPARα (Affinity, Changzhou, China), PGC-1α (Proteintech, Wuhan, China), GAPDH (Proteintech, Wuhan, China), and Blots were analysed using chemiluminescence with imaging system (Bio-Rad, CA, USA).

### Statistical analysis

All data were expressed as mean ± SD, and analysed using Student’s *t*-test or one-way analysis of variance, followed by Tukey’s test. GraphPad Prism 7.0 software (GraphPad, Inc., La Jolla, CA, USA) was used, and values of *p* < 0.05 were considered statistically significant.

## Results

### TW induced liver injury in C57BL/6J mice

**Figure 2.**
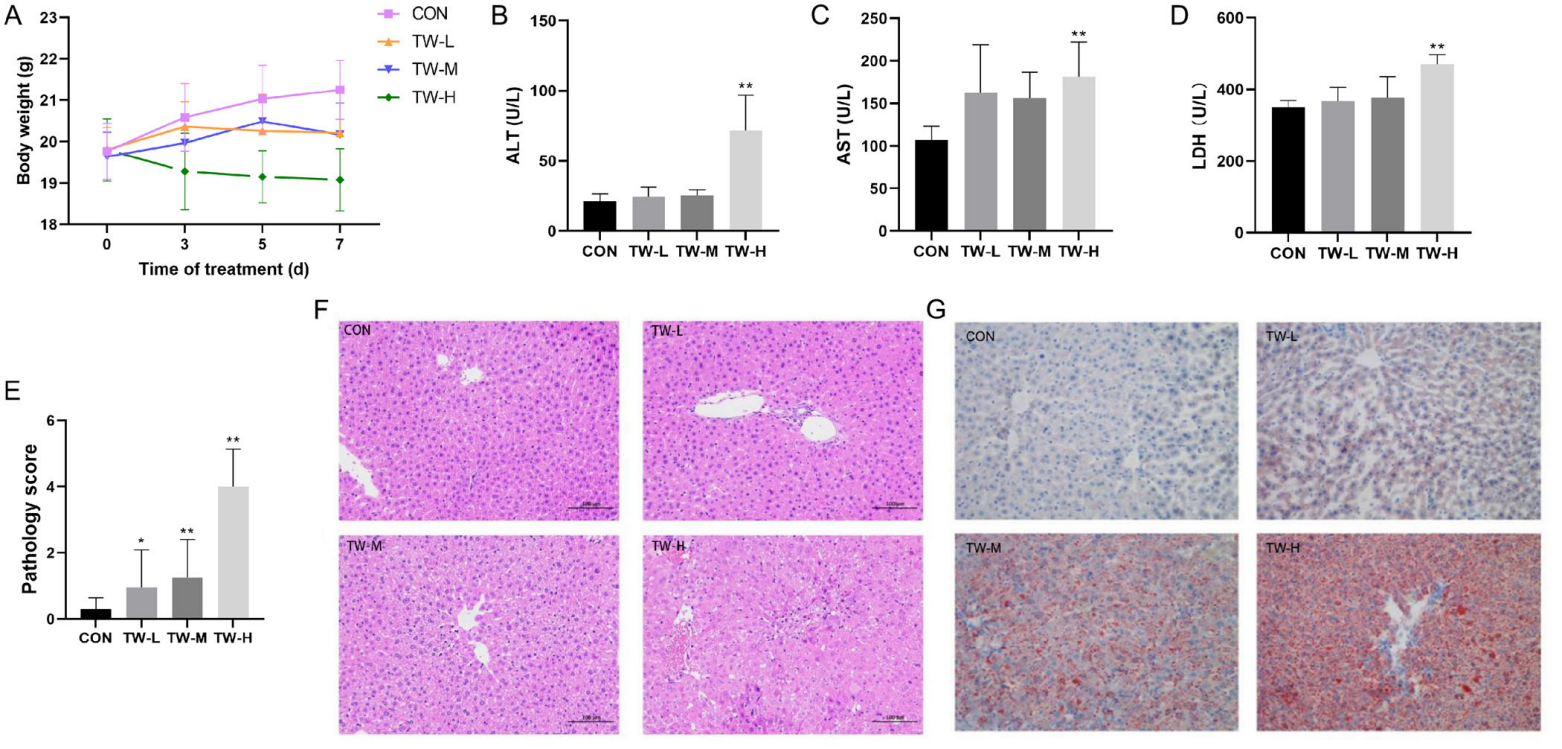
*Tripterygium wilfordii* (TW)-induced liver injury in C57BL/6J mice. (A) Body weight changes at indicated time points. (B–D) Serum ALT, AST and LDH levels. (E, F) Hematoxylin-eosin (H&E) staining and pathology score of liver tissue. (G) Oil red O (ORO) staining. Data are presented as mean ± SD (*n* = 10). *p < 0.05, **p < 0.01 vs. control group.

TW dose-dependently reduced body weight and elevated serum ALT, AST, and LDH levels in mice (Figure 2(A–D)). Histological analysis revealed that TW caused hepatocellular injury in the form of cellular oedema, inflammatory cell infiltration, and cellular steatosis (Figure 2(E,F)). Oil red O staining confirmed the increased lipid droplets in the liver tissue of TW-treated mice (Figure 2(G)). The above results indicate that TW hepatotoxicity is dose-dependent and can perturb hepatic lipid metabolism in mice. Because the high-dose TW group caused a significant liver injury in mice, we selected this dose for subsequent experiments.

### QLT protected against TW-induced liver injury and lipotoxicity in C57BL/6J mice

**Figure 3.**
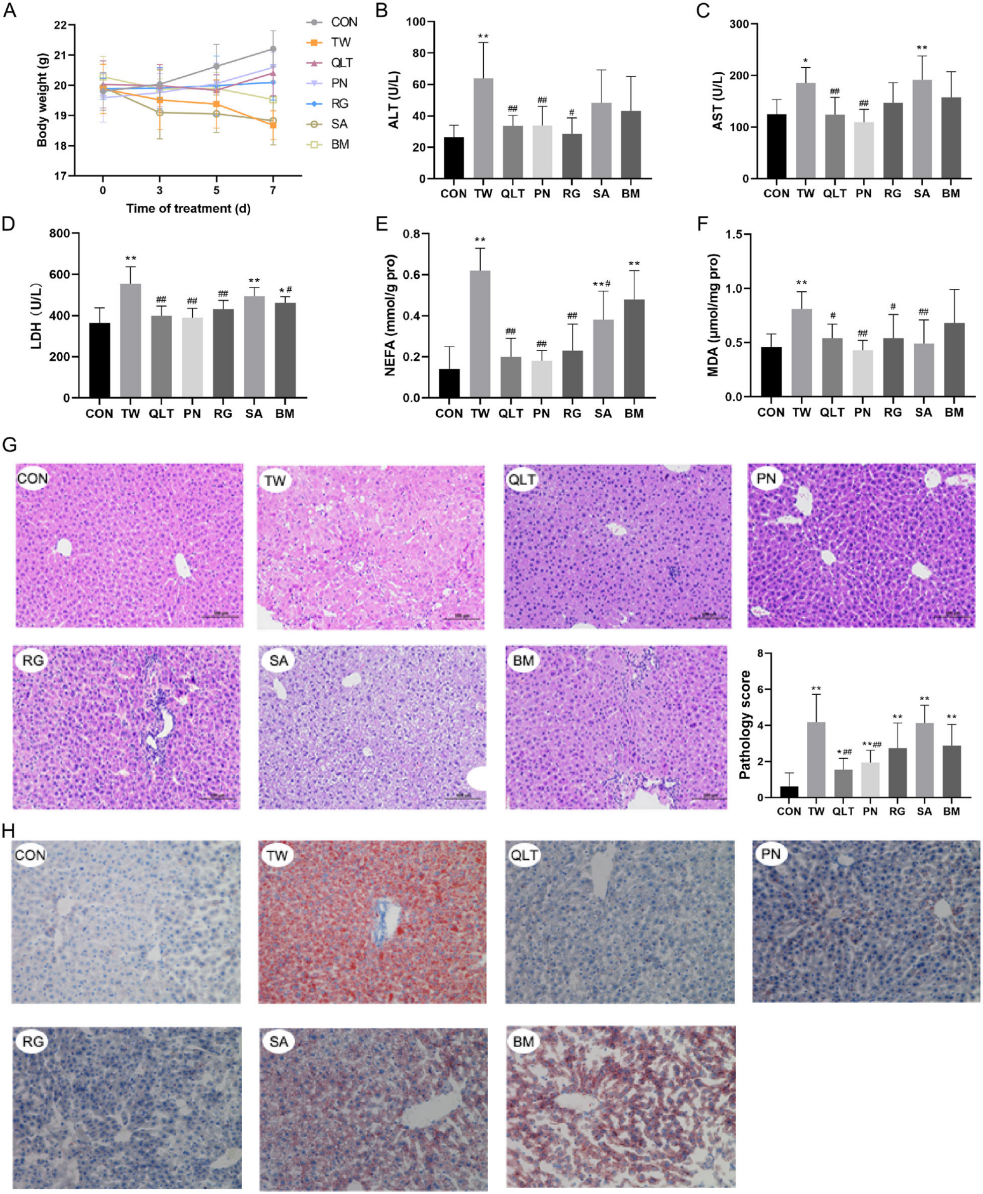
Qingluotongbi formula (QLT) protected against *Tripterygium wilfordii* (TW)-induced liver injury and lipotoxicity in C57BL/6J mice. (A) Body weight changes at indicated time points. (B–D) Serum ALT, AST and LDH levels. (E, F) NEFA and MDA content in liver tissue homogenates. (G) Hematoxylin-eosin (H&E) staining and pathology score of liver tissue. (H) Oil red O (ORO) staining. Data are presented as mean ± SD (*n* = 8). *p < 0.05, **p < 0.01 vs. control, ^#^*p* < 0.05, ^##^*p* < 0.01 vs. TW group.

We then observed the protective effect of QLT and individual QLT ingredients on TW-induced liver injury in mice. The results showed that both QLT and the individual ingredients could alleviate TW-induced liver injury to varying degrees. Among these, QLT, PNS, and CAT exhibited the most significant elevation in mice body weight (Figure 3(A)) and substantial reduction in both serum liver enzyme levels (Figure 3(B–D)) and lipid accumulation (Figure 3(H)). Also, QLT and CAT substantially improved pathology scores (Figure 3(G)). However, the improvement effect was not evident with SA and BM, presumably due to them playing a more important role in terms of therapeutic effect.

Since lipid accumulation and lipid-induced injury are usually associated with increased lipid peroxidation (Malaguarnera et al. 2006), we detected the levels of non-esterified free fatty acids (NEFA) and malonaldehyde (MDA) in mouse liver tissue. Elevated levels of free fatty acids, specifically polyunsaturated fatty acids (PUFAs), can sensitize cells to lipid peroxidation. QLT and the individual ingredients markedly reduced the NEFA and MDA levels, protecting the liver from lipid peroxidation damage (Figure 3(E,F)).

### QLT attenuated TW-induced mitochondrial damage

**Figure 4.**
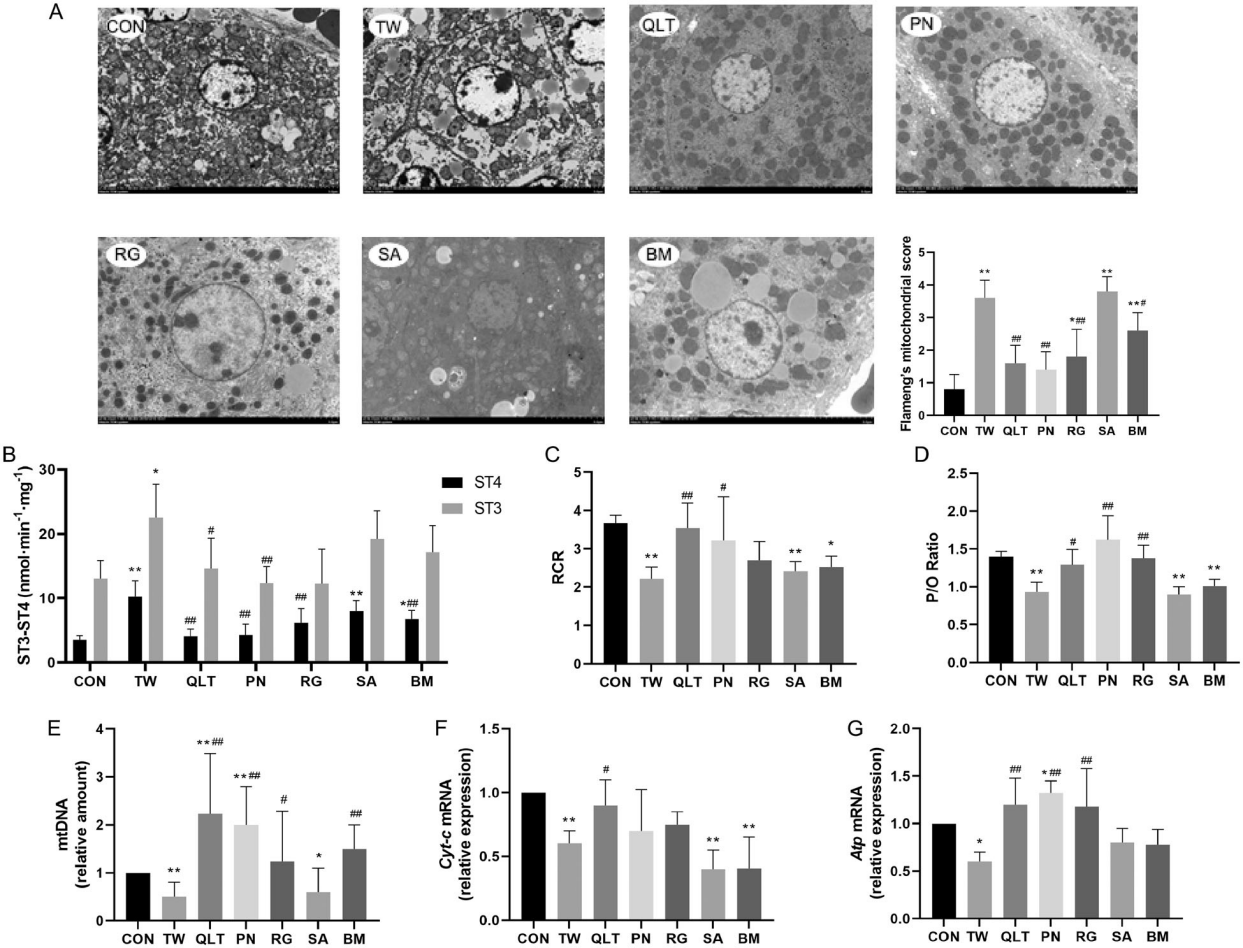
Qingluotongbi formula (QLT) attenuated *Tripterygium wilfordii* (TW)-induced mitochondrial damage. (A) Liver transmission electron micrographs and the Flameng’s mitochondrial score. (B) Mitochondrial state 3 and 4 oxygen consumption. (C, D) The respiratory control rate (RCR) and phosphorus-oxygen ratio (P/O). (E) Relative mt DNA copy number. (F, G) The mRNA levels of Cyt-c and Atp. (A) Data are presented as mean ± SD (*n* = 5). (B–G) Data are presented as mean ± SD (*n* = 8). *p < 0.05, **p < 0.01 vs. control, ^#^*p* < 0.05, ^##^*p* < 0.01 vs. TW group.

Mitochondria are the major sites of cellular fatty acid oxidation and ATP synthesis, and mitochondrial damage is the critical event contributing to drug-induced liver injury. Promoting mitochondrial function and enhancing fatty acid oxidation are considered effective strategies to reduce lipotoxicity (Fromenty and Pessayre 1995). To clarify whether the mechanism of TW hepatotoxicity is related to mitochondrial damage, we observed the ultrastructural changes in the liver. Transmission electron microscopy analyses showed that TW triggered mitochondrial damage in hepatocytes, manifesting as a decreased number of mitochondria, reduction or vanishing of mitochondrial cristae, and the rupture of the outer mitochondrial membrane. QLT and individual QLT ingredients can mitigate mitochondrial impairment induced by TW (Figure 4(A)).

Mitochondrial function is classically defined as the ability to generate ATP through fatty acid β-oxidation and oxidative phosphorylation (VanderVeen et al. 2017). Mitochondrial respiratory control ratio (RCR) and phosphorus-oxygen ratio (P/O) can reflect the coupling degree of mitochondrial respiratory chain to oxidative phosphorylation, and are important indicators for evaluating mitochondrial function. Uncoupling of the mitochondrial respiratory chain leads to excess production of reactive oxygen species (ROS) and induces lipid peroxidative damage (Selivanov et al. 2011). We found TW significantly decreased the mitochondrial RCR and P/O of hepatocytes. QLT and PN substantially increased both RCR and P/O, and RG showed improvement effects on P/O but not RCR (Figure 4(B–D)). mtDNA encodes genes related to the respiratory chain complex. MtDNA damage can directly affect the electron transfer process, reducing ATP production, and causing energy metabolism disorders (Sun et al. 2018). TW group displayed a significant decrease in mtDNA copy number as well as transcriptional levels of Cytochrome-c (Cyt-c) and Atp, the key components of the mitochondrial respiratory chain. Whereas QLT and its ingredients except SA restored the mtDNA copy number, and QLT also increased the transcriptional levels of Cyt-c and Atp (Figure 4(E–G)). Besides, PN and RG elevated transcriptional levels of Atp (Figure 4(G)).

The above results indicate that QLT exerted a protective effect by mitigating mitochondrial damage and promoting mitochondrial function.

### QLT reversed TW-induced suppression of PPARα/PGC-1α pathway

**Figure 5.**
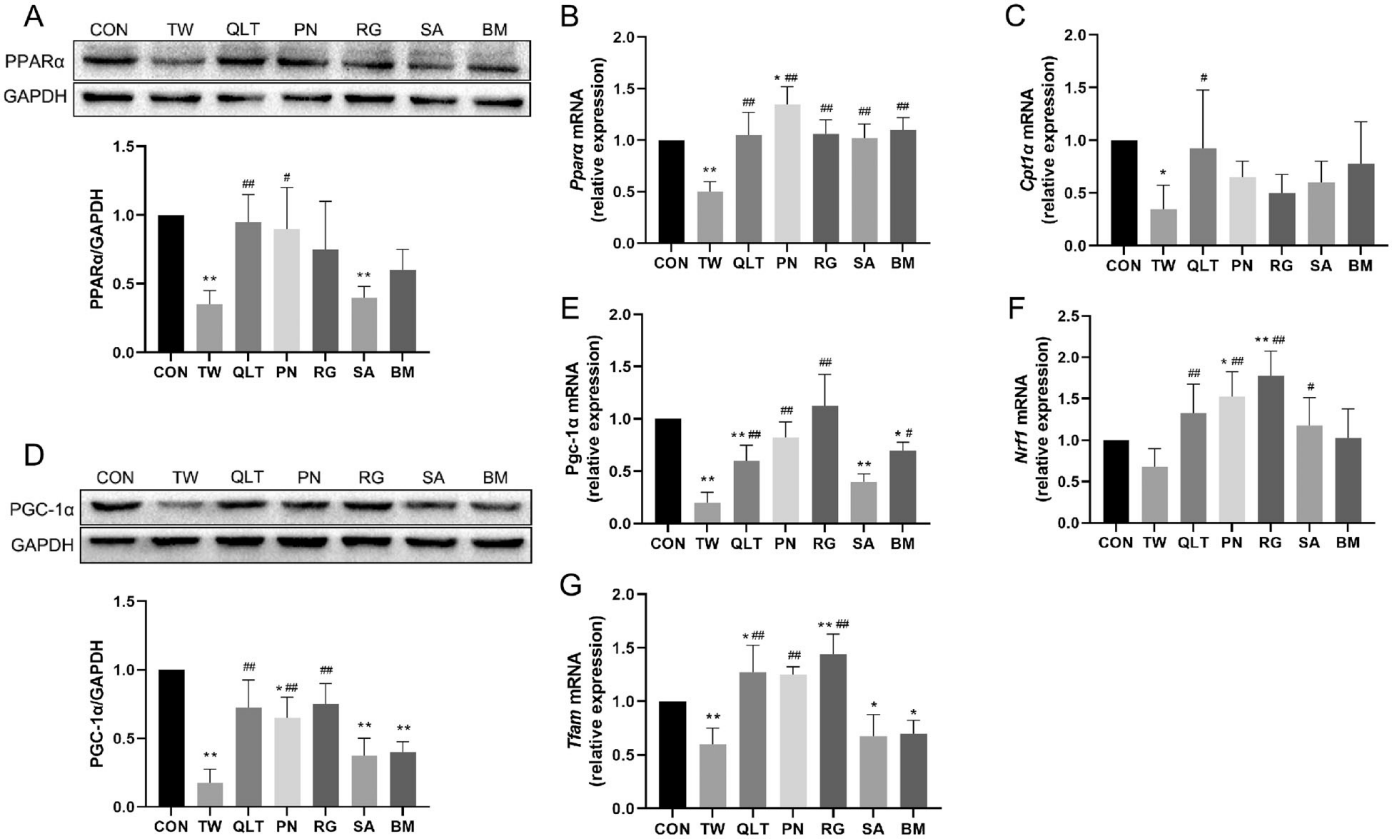
Qingluotongbi formula (QLT) reversed *Tripterygium wilfordii* (TW)-induced suppression of PPARα/PGC-1α pathway. (A) PPARα protein levels in mice liver tissues. (B, C) The mRNA levels of PPARα and Cpt-1α in mice liver tissues. (D) PGC-1α protein levels in mice liver tissues. (E–G) The mRNA levels of Pgc-1α, Tfam and Nrf1 in mice liver tissues. Data are presented as mean ± SD (*n* = 8). *p < 0.05, **p < 0.01 vs. control, ^#^*p* < 0.05, ^##^*p* < 0.01 vs. TW group.

To further elucidate the mechanism, we examined whether TW and QLT can regulate the PPARα/PGC-1α pathway. PPARα is an important nuclear receptor that regulates lipid metabolism, and its downstream enzyme Carnitine *O*-palmitoyltransferase 1 (CPT-1α) can transport fatty acids from the cytoplasm to mitochondria, a rate-limiting step in fatty acid β-oxidation. The results showed that TW significantly down‐regulated the protein expression of PPARα, consistently with down-regulation of Pparα and Cpt-1α gene expression. We found that both QLT and individual QLT ingredients could significantly up-regulate the expression of PPARα at the mRNA level, QLT and PN significantly increased PPARα protein level, and QLT showed the most pronounced up-regulation of Cpt-1α mRNA expression among all groups (Figure 5(A–C)).

PGC-1α governs mitochondrial biogenesis and oxidative phosphorylation by upregulating nuclear respiratory factor 1 (NRF1) and mitochondrial transcription factor A (TFAM). TW significantly suppressed PGC-1α protein expression and down-regulated the mRNA levels of Pgc1-α, Nrf1, and Tfam. QLT, PN and RG promoted PGC-1α protein expression and the gene expression of Pgc1-α and Tfam, and all groups except BM exhibited significant upregulation of Nrf1 mRNA levels (Figure 5(D–G)).

These data suggest that TW can inhibit the PPARα/PGC-1α pathway, QLT and its ingredients can reverse TW-induced inhibition of the PPARα/PGC-1α pathway, and promote downstream target genes transcription, thereby improving mitochondrial function and enhancing fatty acid oxidation.

## Discussion

TW possesses anti-inflammatory and immunomodulatory effects, and has been widely used in the treatment of autoimmune and inflammatory diseases. However, TW is known to have toxic side effects (Song et al. 2020). Drug-induced liver injury (DILI) has become an increasingly important clinical problem (Weaver et al. 2020), and the occurrence probability of DILI in mainland China is higher than that in Western countries as reported, and traditional Chinese medicines were the major causes of DILI in mainland China (Shen et al. 2019). Traditional Chinese medicine-induced liver injury (TILI) has been the main obstacle limiting the clinical application and translation of traditional Chinese medicine. Therefore, it is urgently necessary to understand the underlying mechanisms and find methods to alleviate drug-induced hepatotoxicity.

The compatibility of different traditional Chinese medicines exerts a synergistic effect, and is of proven effectiveness to reduce drug toxicity in clinical application. We have previously found that QLT and its ingredients could restore liver lipid metabolism homeostasis and reduce TW-induced liver injury in SD rats (Yu et al. 2022). In this study, we noted that the protective effects of QLT are closely related to mitochondrial function and fatty acid metabolism. TW-induced mice liver injury occurred with significant lipid accumulation and mitochondrial dysfunction. As the central organ of lipid metabolism, liver lipid metabolism homeostasis is crucial to normal liver function. Impaired fatty acid oxidation contributes to excessive lipid accumulation in the liver, which cannot be metabolic clearance in time will result in steatosis (Morio et al. 2021). Furthermore, increased free fatty acid concentrations can lead to lipotoxicity, causing cellular stress, apoptosis and necrosis (Hirsova et al. 2016). Besides direct cytotoxicity, enhanced insulin resistance that may be induced can result in further hepatic lipid accumulation and trigger inflammatory responses (Machado and Diehl 2016).

Mitochondria are the key organelles involved in cellular lipid metabolism and have been reported to be the targets for various drug-induced hepatotoxicities (Begriche et al. 2011). Studies have shown that mitochondrial damage is one of the main mechanisms of triptolide cytotoxicity, the main active and toxic component of TW (Yao et al. 2008; Fu et al. 2013; Chen et al. 2020). Liver lipid homeostasis can be disrupted by mitochondrial dysfunction, such as inhibited fatty acid oxidation (Natarajan et al. 2006; Chen et al. 2007). The fatty acid oxidation process produces acetyl-CoA, which will be oxidized in the tricarboxylic acid cycle, and provides electrons passed to the respiratory chain for ATP synthesis, ensuring cellular energy supply. There is evidence that mitochondrial fatty acid oxidation and oxidative phosphorylation processes are not only functionally interrelated but also physically connected, which can reduce electron leakage and ROS production during the processes (Wang et al. 2019).

Though modest mitochondrial stress can cause adaptive responses that protect mitochondria and the cell from future stimuli, unresolved stress can be a powerful inducer of cell death (Calabrese et al. 2010; Perluigi et al. 2010). Mitochondrial damage and inhibition of fatty acid oxidation can severely disrupt cellular energy metabolism, which in turn further aggravates mitochondrial dysfunction. This may create a vicious cycle, promoting liver injury.

Mitochondria are fully integrated into the cell. The functional state of mitochondria, is a highly regulated process governed by a complex network of intracellular signalling pathways (Drake et al. 2003; Calabrese et al. 2007). As crucial transcription factors, the interaction of PPARα and PGC-1α coordinates the expression of genes involved in mitochondrial function and lipid metabolism. Thus, restoring mitochondrial metabolic homeostasis is the key to mitigating TW-induced liver injury.

Our results suggest that the compatibility of different ingredients in QLT can improve mitochondrial function and enhance fatty acid β-oxidation to a varying degree compared to the TW group, and the improvement was most obvious in the QLT group. It is reported that RG and PN have protective roles in mitochondrial disorganization by restoring mitochondrial membrane potential, ATP levels, and mtDNA copy number (Li et al. 2014; Yang and Bao 2017). Our previous studies showed that *Panax notoginseng* saponins and catalpol, the active ingredients of RG and PN, reduced mitochondrial damage, inhibited apoptosis, and alleviated triptolide-induced liver injury in SD rats (Zhou et al. 2018). Interestingly, in this study, we found that PN was superior to RG in activating PPARα to promote fatty acid β-oxidation, while RG was better than PN in activating PGC-1α to promote mitochondrial biogenesis. We speculate that they likely play a synergistic role in enhancing PPARα/PGC-1α pathway activation.

Although SA and BM seem to play a less prominent role in protecting against TW-induced liver injury, we found that SA significantly reduced the free fatty acids and lipid peroxidation product MDA in mouse livers, indicating that it may have an advantage in mitigating lipid peroxidation damage. The mitochondrial injury scores decreased and the mtDNA copy number increased significantly in the BM group, suggesting that BM can improve mitochondria structure and function to some extent. Both of them can promote the gene transcription and protein expression related to PPARα/PGC-1α pathway. Collectively, different ingredients of QLT had different functional roles in the PPARα/PGC-1α pathway to regulate mitochondrial function and liver lipid metabolism, synergistically attenuating TW hepatotoxicity.

## Conclusions

Our results suggest that TW-induced liver injury is associated with mitochondrial damage and lipid metabolism disorders. TW suppresses the PPARα/PGC-1α pathway and impairs mitochondrial function, thus leading to lipid metabolism disorders and injury in mouse livers. QLT reduces the hepatotoxicity of TW by activating the PPARα/PGC-1α pathway, promoting mitochondrial biogenesis and enhancing fatty acid β-oxidation. Our study contributes to further understanding the underlying mechanism of QLT’s protective effect against TW hepatotoxicity, providing a reference for the safety of TW in clinical applications.
